# The federated trials approach; an opportunity for global collaboration in health emergencies

**DOI:** 10.1016/j.eclinm.2026.103809

**Published:** 2026-02-25

**Authors:** Inge Christoffer Olsen, Lu Zheng, Skerdi Haviari, Alain Amstutz, Yazdan Yazdanpanah, Franz König, Thomas R. Fleming, Matthias Briel, UNITY/MPX-RESPONSE Study Group, Beatriz Grinsztejn, Valdiléa G. Veloso, Sandra Wagner Cardoso, Mayara Secco Torres da Silva, Maria Pia Diniz, José Valdez Ramalho Madruga, Jorge Andrade Pinto, Esaú Custódio João, Luiz Carlos Pereira Junior, José Henrique Pilotto, Carlos Brites, Maria Paula Gomes Mourão, Alexandra Calmy, Maxime Hentzien, Olivier Segeral, Stefano Musumeci, Laurence Toutous-Trellu, Sabine Yerly, Benjamin Hampel, Pedro Enrique Cahn, Maria José Rolón, Dimie Ogoina, Miguel Ekkelenkamp, Nathalie Strub-Wourgaft, Ines Aristegui, Thiago Torres, Osilade Adewole, Roger Lewis, Yazdan Yazdanpanah, France Mentré, Axelle Dupont, Najeh Daabek, Inge Christoffer Olsen, Skerdi Haviari, Cedric Laueuenan, Alain Amstutz, Matthias Briel, Constance Delaugerre, Xavier de Lamballerie, Gilles Peytavin, Alpha Diallo, Léa Vitu, Mario Delgado-Ortega, Erica Telford, Keyla de Almeida Macedo, Roberta Trefiglio, Laetitia Guiraud, Luciana Gambardella, Carolina Perez, Ventzislava Petrov-Sanchez, Michèle Genin, Maelle Coupez, Isabelle Hoffmann, Cecilie Moe, Maria Figueroa, STOMP Study Group, Timothy Wilkin, William Fischer, Jason Zucker, Lara Hosey, Jhoanna Roa, Arzhang Javan, John Brooks, Judith Currier, Joseph Eron, Rajesh Gandhi, Matthew Hamill, Kieron Leslie, Sharon Nachman, Caitlyn McCarthy, Carlee Moser, Justin Ritz, Pooja Saha, Lu (Summer) Zheng, Stephanie Caruso, Caroline Reeb, Shahadah Bailey, Lauren Mabe, Lisette Molins, Sujith Valiyaparambil, Grace Aldrovandi, Kathie Ferbas, Faye Landsman, Jade Paris, Danielle Campbell, Stanford Chimutimunzeve, Kristina Brooks, Edmund Capparelli, Shawn Chiambah, Jonathan Berardi, Alexander L. Greninger, Christine Johnston, David Smith, Cheryl Day, Emily Blum, Josie Marshall, Jillian Laroche, Bridget Makhlouf

**Affiliations:** a Department of Research Support for Clinical Trials, Oslo University Hospital, Oslo, Norway; b Department of Biostatistics, Harvard T.H. Chan School of Public Health, Boston, MA, USA; c Université Paris Cité, Inserm, IAME, Paris, 75018, France; d CLEAR Methods Center, Division of Clinical Epidemiology, Department Clinical Research, University Hospital Basel and University of Basel, Basel, Switzerland; e Population Health Sciences, Bristol Medical School, University of Bristol, UK; f Service de Maladies Infectieuses et Tropicales, Hôpital Bichat, AP-HP, Paris, France; g Institute of Medical Statistics, Center for Medical Data Science, Medical University of Vienna, Vienna, Austria; h Department of Biostatistics, University of Washington, Seattle, WA, USA

**Keywords:** Federated trials, Public health emergencies, Conjoined analysis, Data monitoring committee, Regulatory decision-making

## Abstract

The gold standard for providing confirmatory evidence to regulatory agencies is a single sponsor conducting a randomised, controlled clinical trial (RCT). But emerging situations like the mpox outbreak can complicate launching large, single, global trials that meet the needs of multiple stakeholders, including multinational funders and regulators.

Drawing on lessons from the mpox outbreak, we propose an alternative approach: the federated trial design. This approach ensures that individual trials launch quickly and that a rigorous, prespecified, conjoined analysis using combined data supports joint regulatory decisions. Early engagement with regulatory agencies is crucial to arranging such a conjoined analysis.

Federating trials can support regulatory decision-making when they include a prespecified conjoined analysis that is sufficiently rigorous. Essential steps include harmonising individual trial protocols, aligning data standards, and arranging for a single Data Monitoring Committee to review a combined, multi-trial analysis.

The classical single-trial approach remains the gold standard, but investigators should consider federated trials in emergencies that complicate conducting single trials. In such crises, investigators need to explain clearly why standalone evidence from participating RCTs is not obtainable. The federated trials design cannot replace the classical design, but can provide timely, robust evidence in crises such as public health emergencies.

## Introduction

Mpox is an infectious disease caused by the mpox virus, an orthopoxvirus first identified in humans in 1970 in the Democratic Republic of Congo.^1^ In May 2022 clade IIb mpox spread from central Africa, leading to a global outbreak. The World Health Organisation (WHO) declared the epidemic a public health emergency of international concern in July 2022.^2^ The WHO also issued a call to assess the use of antivirals to treat mpox cases, and to develop a CORE protocol for an adaptive, multiregional, global, randomised, placebo-controlled trial to evaluate the safety and efficacy of drugs for mpox.^3^ Here, ‘adaptive’ refers to adaptive monitoring (i.e., interim analyses allowing for early stopping either for futility or efficacy), rather than adaptive platform or response-adaptive designs. An international group of experts wrote the CORE protocol based on the PALM 007 protocol (NCT05559099), a trial sponsored by the U.S. National Institute of Allergy and Infectious Diseases (NIAID) to evaluate the antiviral tecovirimat for mpox.^4^

The WHO and the CORE protocol called for a single global trial, but several trials emerged in different regions, all assessing the antiviral drug tecovirimat.

• In the U.S., NIAID sponsored the ACTG A5418 trial (STOMP, NCT05534984).^5^
• In the U.K., the University of Oxford sponsored the PLATINUM trial (NCT05534165).^6^
• The European Commission funded a consortium (MPX-RESPONSE) that proposed three daughter trials:
  ○ In Europe, ECRAID sponsored the EPOXI trial (NCT06156566),^7^
  ○ In Brazil, Argentina, and Switzerland, ANRS MIE sponsored the UNITY trial (NCT05597735),^8^ and
  ○ In Central Africa, PANTHER sponsored the MOSA trial.^9^

**Fig. 1:**
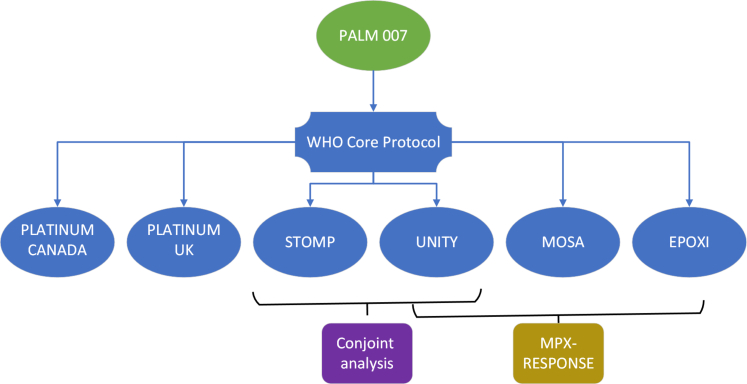
Overview of the federated structure of the MPX-RESPONSE consortium and related mpox trials.

**Fig. 2:**
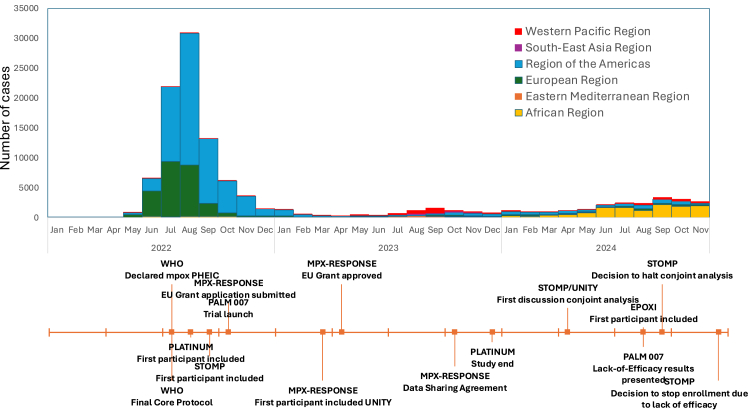
Monthly global incidence of mpox and key milestones for the tecovirimat trials.

Fig. 1 illustrates the relationship between these trials. Fig. 2 presents the monthly global incidence of mpox and a milestone timeline for the trials.

Multiple factors prevented funders from establishing a single, unified global trial. The WHO and the CORE Protocol authors had envisioned such a trial, but the varying legal and regulatory frameworks, funding sources, and institutional mandates the collaborating partners faced rendered that vision infeasible. For example, governance and operational constraints, not scientific considerations, prompted the MPX-RESPONSE Consortium to develop three separate trials and protocols rather than a single, multinational study. The Consortium aimed to harmonise trial infrastructure and have investigators share data prospectively across trials. Crucial to this aim was establishing a common Data Monitoring Committee (DMC) with access to pooled, unblinded data. That DMC would rely on a shared-data infrastructure that enabled near real-time monitoring. Sharing information, governance, and material resources is an essential feature of federated trial design.

From 2020 to 2022, the COVID-19 pandemic illustrated the consequences of fragmented clinical research in public health emergencies. Hundreds of separate trials launched in parallel, many too small or heterogeneous to yield definitive conclusions. Those trials thus exposed volunteers to studies unlikely to produce meaningful evidence. Design weaknesses in those trials cost investigators time when identifying effective interventions was urgent. These problems underscored the need for international stakeholders to coordinate approaches and prospectively harmonise trial protocols.

We describe federated trial design elements that can help investigators overcome obstacles to implementing a classic single trial, such as complex regulations, funding, or operations. Investigators need to weigh such obstacles against the scientific value of a single-trial design. We also outline a joint decision-making process in federated trials and the essential steps in performing an analysis of a confirmatory trial intended to support regulatory decision making without a large, international, multicentre trial. We use the MPX-RESPONSE consortium to illustrate the federated trials approach and discuss the planned but unrealised conjoined analysis between the UNITY and STOMP trials (the STOMP trial ended prematurely after an interim futility analysis showed that tecovirimat was safe but did not lead to faster resolution of mpox lesions or improved pain control).^10^ Together, these examples highlight the key methodological and analytical factors in applying a federated framework to generate regulatory-grade evidence. Finally, we discuss our experiences in the context of preparing for the next pandemic.

## The federated trials design

The classic design for a pivotal clinical trial is a single trial, usually with a single sponsor.^11^ This structure reflects regulatory and operational considerations, as a single sponsor ensures consistent governance, monitoring, data management, and legal accountability across all participating sites. But multi-sponsor trials, such as cosponsored trials under the EU Clinical Trials Regulation, involve varying responsibilities and oversight that can complicate trial conduct and regulatory compliance.^12^ A single, well-controlled trial therefore remains the standard for pivotal evidence, as it promotes consistency and clear regulatory interactions. But political, financial, or logistical constraints may prevent this optimum, and alternative designs can help generate timely, robust evidence in global health emergencies that require simultaneous efforts across regions.^13^^,^^14^

The federated trials design prospectively sets up separate, independent trials while planning to combine data from those trials to answer common clinical research questions to support regulatory decision making.^15^ This design helps investigators overcome regulatory, budgetary and operational hurdles, such as obtaining trial authorisation and starting the individual trials faster than under the standard, single-trial, single-protocol approach. Running multi-national, multi-regional intervention trials typically requires pharmaceutical companies or large academic or government organisations, such as the US National Institutes of Health, the UK National Health Service or the ANRS MIE/INSERM in France. This structure limits the capacity to conduct trials of public interest or in global health crises that require rapid responses. The federated trials design aims to remedy these limitations of the classic single-trial design by assigning responsibility for organising individual trials, managing workloads and securing funding among several local sponsors. Assignments may be geographical, e.g., by country or continent, which can increase local ownership of individual trials. That ownership ensures investigators can apply their local regulatory knowledge and can motivate study personnel to feel they are part of a collaborative venture and not merely serving one global trial sponsor. Similar clinical trial designs aim to prospectively combine information from different trials, e.g., prospective meta-analyses or meta-trials. But such studies often rely on finding trials already underway, which have distinct protocols, so often preventing the larger project from meeting regulatory requirements (Table 1).^16^, ^17^, ^18^, ^19^, ^20^

**Table 1:** The federated trials design compared to other similar designs.

	Individual trials followed by a retrospective meta-analysis	International single trial	Meta-trial: prospective international meta-analysis	Federated trials design
Identification of trials	During trial or after completion	Not applicable	Before initiation or during trial	Preferably before initiation or also possible during trial
Eligibility criteria for participants	Heterogenous between trials	Uniform within the trial	Similar between trials (amendments of individual trial protocols possible before trial initiation)	Similar, or based on same CORE protocol. Eligibility criteria for the conjoined analysis prespecified.
Intervention details and how they were administered	Heterogenous between trials	Uniform within the trial	Uniform between trials: agreement between individual investigators to deliver same intervention	Uniform, or based on the CORE protocol
Pre-specified primary and secondary outcome measures	Heterogenous between trials	Uniform within the trial	Uniform between trials (investigators agree on a common set of outcomes)	Uniform and/or based on the CORE protocol
Samples size and Interim analysis	Heterogenous between trials, interim analyses impossible at the meta-level	One sample size calculation for the trial, interim analyses possible	Meta-trial design transcends original sample size calculation, interim analyses possible at the meta-level	Described independently but consistently in each protocol. Clear and prospective description on the combined sample size and interim analyses.
Randomisation- sequence generation, stratification, allocation sequence, concealment and blinding	Heterogenous between trials	Centralised randomisation	May differ for each site but fundamental randomisation principles adhered to	Based on the CORE protocol, but adapted to each trial
Statistical methods	Heterogenous original analyses, meta-analysis on effect sizes to compute a summary effect	Uniform within the trial, adjustments possible	Uniform within the trials, meta-analysis on individual participant data, adjustments possible	Uniform across the trials, conjoined analysis on individual participant data, adjustments possible
Analysis populations: intention to treat, Per protocol, subgroups	Heterogenous between trials	Uniform within the trial	Uniform between the trials (agreement on uniform analysis population)	Uniform across the trials (agreement on uniform analysis population)
Data quality and safety monitoring	Each trialist is responsible for his or her trial	Centralised data monitoring	Each trialist is responsible for his or her trial	Each trialist is responsible for his or her trial, but with a common data safety monitoring board
Funding	Multiple funding	Centralised funding	Multiple funding	Multiple funding
Set-up time	Short	Long	Short	Short
Time to completion	Short	Long	Short	Short
Protocols	Multiple original protocols	One	Multiple original protocols followed by a meta-trial protocol	Multiple original protocols followed by a conjoined analysis plan, conjoined analysis prespecified in each of the protocols or added in amendments
Ethics	Each trialist is responsible for his or her trial	Centralised submission process	Each trialist is responsible for his or her trial	Each trialist is responsible for his or her trial

The designs of both the federated MPX-RESPONSE trials and meta-trials implemented in the COVID-19 pandemic aim to assess the efficacy of novel drugs against placebo for marketing authorisation applications. The respective study protocols thus reflected and were authorised by national competent authorities and ethics committees for this purpose. But the expected EPOXI trial sample size lacked sufficient statistical power, making the federated trial design essential to obtaining the required authorisation to initiate the trial. The greater statistical power of combined data makes the federated trial design more attractive in emergencies. An individual, single trial with a single sponsor can launch quickly, but with uncertainty about whether its statistical power will be sufficient.

A central design feature of federated trials is a common DMC, which can perform the most comprehensive safety assessments possible, safeguarding the interests of trial participants. A unified DMC can perform interim efficacy analyses when necessary. Performing conjoined analyses as part of federated trials is also a major advantage over individual trials, which are inherently underpowered for interim efficacy analyses. Trial protocols and DMC charters need to specify conjoined interim analyses. A common DMC can issue its recommendations as a trial-specific DMC would, and the Trial Steering Committee (TSC) can decide to stop or continue a trial, issuing the appropriate clear instructions when recommending that a trial stop. Interim efficacy analyses in federated trials require later adjustments to control type I error rates at predefined nominal levels. Membership in a common DMC requires sufficient experience to interpret results across trials. Harmonised governing documents and clear communication between trial teams are also essential to help prevent TSCs from receiving different recommendations and issuing different decisions. But all trials under a federated design require appropriate firewalls to protect trial integrity and their ongoing conduct by preventing leakage of interim data.

Establishing a shared DMC across independently sponsored trials poses practical and financial challenges. The MPX-RESPONSE consortium received dedicated funds to support the work of the DMC statistician and the independent statistician who performed unblinded data analyses. Such an arrangement may be necessary for federated frameworks to function effectively.

**Fig. 3:**
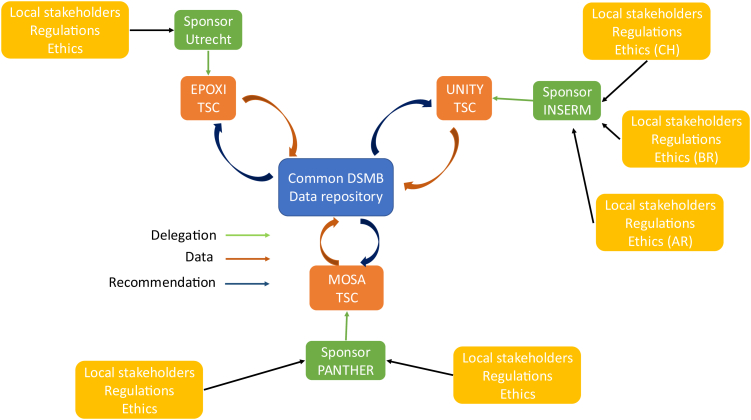
Common Data Safety Monitoring Board structure of the MPX-RESPONSE consortium; DSBM = Data Safety Monitoring Board; TSC = Trial Steering committee; CH = Switzerland, BR = Brazil, AR = Argentina.
• Drafting individual trial protocols based on the WHO CORE protocol and emphasising consistency across trials.
• Adopting a common case report form and data capture solution for the UNITY trial, both of which the MOSA trial then adopted. EPOXI shared features including variable names and structure with UNITY and MOSA.
• Agreeing to do conjoined analyses, i.e., primary analysis for all trials would use the combined data.

The MPX-RESPONSE consortium applied these five practices to its three trials:

• Giving designated investigators and statisticians from all participating trials equal access to a common, regularly updated repository of blinded data.
• Giving a common DMC access to shared unblinded data it could use to inform individual TSCs (see Fig. 3 and the joined DMC charter in the Appendix).

It is vital for federated trial designs to give trial teams the ability to assess specific endpoints and/or estimands, which can vary across trials. Regulators may request that a trial use estimands that are different from those in federated trials but better suited to the epidemiology or regulatory setting of that study population. Each trial team needs the ability to do its own prespecified analysis on pooled data. But these analyses require care to identify and resolve any multiplicity issues resulting from differences in estimands and/or endpoints across trials. Notably, differences in primary endpoints or primary analyses can also arise under the single-trial design, as regulators may apply requirements specific to their regions. Avoiding multiplicity issues thus requires trial teams to prespecify analyses they will perform and ensure that these are clearly reflected in the authorised trial protocols.

In the MPX-RESPONSE project, UNITY and EPOXI shared a primary analysis assessing the same primary estimand, but MOSA prespecified a Bayesian framework to provide estimands more relevant to African participants. Each trial should also generally have flexibility to determine secondary and exploratory endpoints, using combined data, so trial teams can invest in a common effort and still answer questions that interest them.

## Conjoined analysis

Using meta-analytical approaches is an integral part of evidence-based medicine and usually the last stage in developing clinical interventions, both pharmaceutical and non-pharmaceutical. Such evidence synthesis normally uses one of two approaches. A meta-analysis (MA) combines aggregated information from each trial for analysis. An individual participant data meta-analysis (IPDMA) gathers and pools data from each participant in individual trials and conducts consistent analyses on that pooled data. Analyses under both approaches are usually secondary, and thus not intended for regulatory assessment. We define a primary analysis as the analysis that aims to provide pivotal evidence for the primary clinical questions of each trial. The primary analysis thus requires the strict conditions and a strong control for the probability of making a false discovery (the Type 1 error rate). We define a secondary or supportive analysis as any analysis on the same data occurring after the primary analysis. In the drug development paradigm, regulatory assessments for marketing authorisations usually rely on primary analyses, and rarely on secondary analyses such as meta-analyses (either standard or based on individual participant data). The U.S. FDA considers meta-analyses mostly useful for safety outcomes rather than efficacy ones. The FDA also recommends conducting regulatory meta-analyses on trials selected without knowing their results, which is often impossible unless the meta-analysis is prospective.^21^ The European Medicines Agency (EMA) broadly concurs with this guidance, noting that meta-analyses are weaker than one really robust trial supported by smaller trials, and citing a lack of pre-specification, study identification, and heterogeneity as major issues.^22^ One strength of the federated trials approach is that it can effectively address these concerns.

As we clarified earlier, the MPX-RESPONSE consortium is a prime example of a fully federated framework, whereas the planned UNITY/STOMP collaboration reflected a later, more limited application of federated trial principles. The UNITY and STOMP trials launched independently and thus did not constitute a strictly federated trial, but the coordinated efforts of the two study teams showed how to apply elements of the federated approach retrospectively, such as harmonising protocols, arranging shared oversight, and engaging in early regulatory dialogue. In contrast, the EPOXI, MOSA and UNITY trials within MPX-RESPONSE were prospectively coordinated under a shared governance structure, common data infrastructure, and single DMC, exemplifying a true federated design.

## Elements of conjoined analyses

Performing a conjoined analysis involves a series of interrelated parts that can progress concurrently.

### Assessing feasibility and suitability

Investigators first need to identify potential networks or projects and evaluate the feasibility and scientific rationale for doing a conjoined analysis. The requirements for performing a conjoined analysis are much stricter than a regular prospective meta-analysis, as the aim of the former is to provide conclusive evidence with strong false positive discovery rate control. The most important requirement is consistency across protocols, especially for eligibility criteria, intervention, control regimen and outcome, but also about how to gather safety information and perform statistical analyses. Equally vital to ensuring a conjoined analysis yields reliable results is when investigators decide to conduct a conjoined analysis and what information is then available. Making this decision while planning all individual trials raises fewer regulatory concerns than agreeing to conduct a conjoined analysis when some trials are underway or interim analyses have already occurred. Knowing some results could introduce a kind of selection bias when deciding which trials to include in a conjoined analysis. An earlier decision also helps protocol authors harmonise the text across protocols for all trials.

A conjoined analysis is the most natural course when all federated trials are based the same core protocol, but still requires a thorough comparison of individual trial protocols. Small differences across protocols can result from changes regulators request during protocol review and authorisation, or from trial protocol teams interpretating provisions of the core protocol differently. Investigators therefore need to examine and manage such differences before proceeding with federated trials.

The mpox case: Table 2 presents examples of differences between protocols and how to handle them.

**Table 2:** Examples of differences between trials and how to handle them.

Issue	Solutions	Example
Different eligibility criteria	• Defining a restricted population for the conjoined analysis where participants in one or more of the trials are excluded from the analysis population, or • Adjusting for the difference in the statistical analysis, e.g., if there is a difference in the age of the participants then age could be included as a continuous covariate in the primary model	In the STOMP/UNITY conjoined analysis we agreed to exclude from UNITY the participants with a time from treatment onset of more than 14 days, as this was an exclusion criterion in STOMP
Different treatment regimens	• Small differences in treatment schedule are expected and could be handled by including trial as an adjustment factor in the statistical model, either as a fixed or random variable depending on the number of trials included. If the differences are greater, a more formal process should be undertaken by defining the proper estimand and then treating differences as intercurrent events with corresponding handling in the statistical analysis. • Major differences in dosing between the trials could pose a significant challenge as the dose as effect modifier would be confounded by other differences between the trials. Even if a conjoined analysis is not considering the different dosing (just assessing treatment vs control) and then shows benefit of the treatment, the regulators are faced with the challenge of assessing which dose to be used for the indication. So, differences in dosing might render a conjoined analysis infeasible.	In the STOMP/UNITY conjoined analysis there is a difference in the treatment regimen: STOMP trial participants progressing to severe disease during randomised treatment could be switched to open label treatment. This was not the case for UNITY. There were several possible solutions. One was to handle this intercurrent event using a composite outcome strategy in the estimand, defining these subjects as not having reached the primary endpoint of lesion resolution within 28 days
Differences in control conditions	This might be the case when conjoining open-label trials, or when some of the trials are open-label and some are placebo-controlled. Such differences are difficult to handle, and must be carefully considered case by case, preferably in close collaboration with the regulators. But combining placebo-controlled and open-label trials certainly would give the opportunity to assess the impact of the true placebo-effect on the efficacy of the treatment.	Not applicable as all trials on tecovirimat for mpox were placebo controlled
Differences in outcome	The gathering of data should be similar between the trials, but there might be differences in the definitions of the primary endpoint(s). This can be handled by either agreeing on the same primary endpoint, or by defining multiple primary endpoints in the conjoined analysis, with a proper method for multiplicity adjustment.	The STOMP trial had time to active lesion resolution, while UNITY had time to complete lesion resolution as primary endpoint. For the conjoined analysis we decided to have both endpoints as primary, to be tested hierarchically. This was possible because the other endpoint was a secondary endpoint in both protocols.
Differences in analysis and estimands	Even if the primary endpoint is identical there might be differences in the definition of the estimand and the statistical analysis. While details can be handled in the conjoined statistical analysis plan, bigger differences must be sorted out possibly by planning to amend the protocols as part of the trial authorisation process.	See Table 3 for a complete consideration of differences in the primary estimand of STOMP/UNITY

Investigators considering whether to conduct a conjoined analysis weigh two opposing factors: timeliness versus robustness. Conjoined analyses can shorten trials considerably, especially in epidemics, when inclusion rates can vary greatly. Two or more trials that run to their planned completion can provide more robust evidence of the treatment effect than a conclusion based on a single, conjoined analysis. This framework also applies to the question of whether a marketing authorisation requires two pivotal trials.^22^ Investigators also need to consider if conducting a standard retrospective meta-analysis after the trials are complete (including trials that did not participate in a conjoined analysis) will yield better, more robust estimates of investigational treatment efficacy.

The mpox case: The STOMP and UNITY investigators first estimated that conducting a conjoined analysis would save 12–18 months compared to letting either trial to run to completion. The basis for that estimate was assuming each trial planned to recruit about 500 participants and that a conjoined analysis would include 500 participants from the two trials. The investigators discussed two questions, first whether either trial would continue after reaching the target sample size for the conjoined analysis and try to address secondary research questions. A second discussion addressed whether the conjoined analysis would be the only analysis and whether to stop recruitment after reaching the combined sample size of 500 participants. But significantly higher recruitment in both trials prompted the investigators to reduce their estimate of time a conjoined analysis would save to 6 months. They concluded this gain was too small to compensate for the opportunity to obtain robust results from two independent trials running to conclusion. UNITY and STOMP therefore did not implement a conjoined analysis. Nonetheless, the efforts to collaborate and harmonise the two trials yielded a strong basis for conducting an IPDMA of all clade II tecovirimat trials.^23^

### Regulatory communication and protocol harmonisation

Better treatments for patients require obtaining either market authorisation of an investigated treatment or expansion of its indications. Investigators therefore need to communicate clearly and openly with regulatory authorities before conducting a conjoined analysis. Regulators can provide formal or informal advice when assessing protocol amendments. A conjoined analysis will be the primary analysis for the trial, and the basis for making conclusions across all federated trials. Regulators must therefore be satisfied with the analysis methods and the methodology for combining data, especially as conjoined analysis is novel with little precedent from prior regulatory assessments. Authors of federated trial protocols need to identify differences between those protocols, resolve them and ensure a clear, prospective analysis plan describing the conjoined analysis appears in all protocols. Authors should also update the required sample size when necessary and include a joint sample size calculation. Including the conjoined analysis from inception as an option for the primary analysis, especially during pandemics, when flexibility is important.

The mpox case: In UNITY we had several meetings with the EMA Emergency Task Force to handle regulatory activities in preparation for and during a public-health emergency. The STOMP/UNITY teams jointly prepared specifications of the conjoined analysis, then submitted them to the FDA and included them in the UNITY protocol for regulatory assessment. Regulators responded to this submission acknowledging the conjoined analysis, but stating their preference for running the trials to conclusion for a more robust evaluation of tecovirimat efficacy. The FDA also required adherence to the original estimand from the STOMP protocol (based on the competing risk methodology and the Fine–Grey estimator), pointing to the challenges of doing conjoined analyses in a regulatory setting.

### Forming a collaboration

A third crucial early step in federated trials is forming formal and informal collaborations. Investigators from different trials need to agree they will keep each other informed and draft an agreement that details their collaboration.

The MPX-RESPONSE project investigators formalised its collaboration in a consortium agreement. The UNITY and STOMP TSCs agreed to collaborate closely, providing each other updates on study progress and events, and holding multiple meetings to plan the conjoined analysis. The UNITY and STOMP DMCs also made contact and confidentially discussed implications of events.

### Data sharing and management

The consortium agreement should cover critical aspects of collaboration, such as.

• How, where, and when the parties will make data accessible and share them for analysis
• How to identify differences in monitoring and data quality control across trials so all parties will understand them
• How to assess and mitigate potential risks resulting from variable data quality, harmonising monitoring plans and quality assurance procedures whenever feasible.

The mpox case: In the MPX-RESPONSE consortium we set up a data-sharing repository housing blinded data from all trials. We could thus use this repository to combine and harmonise data and prepare to analyse it before unblinding. We decided all trials should follow the Clinical Data Interchange Standard Consortium (CDISC) standards, specifically the Standard Data Tabulation Model (SDTM) and the Analysis Data Model (ADaM) to facilitate the data integration and subsequent unblinding. The data from each trial was then sent regularly to a secured data repository at Oslo University Hospital.

### Statistical analysis plan and analyses

The investigators should start developing the conjoined statistical analysis plan (SAP) when planning a federated trial, and consult regulators as they proceed. The SAP must meet all regulatory requirements, especially on controlling for the false discovery rate. SAP authors need to consider any alpha spent in individual trials when they define the hypothesis testing regime. The SAP must both specify a common estimand and resolve any discrepancies in the primary estimand. It must also include a clear plan for the joint sample size and specify the conclusions based on the conjoined analysis. Prespecifying a conjoined analysis helps investigators conduct a structured assessment of treatment-effect heterogeneity across trials. Hierarchical models, whether frequentist or Bayesian, can quantify between-trial variability, improving interpretability of combined results.^24^

The mpox case: We discussed in detail how to handle discrepancies in the STOMP/UNITY conjoined analysis. The resulting estimand table appears in Table 3. Our conjoined analysis also had to account for STOMP having spent a small amount of alpha on a previous efficacy interim analysis. We therefore decided to simply subtract that amount from the final analysis. But researchers should develop a more flexible framework in the future to address this *partial* alpha spending situation. Finally, we determined the STOMP/UNITY sample size would be the larger of the individual sample sizes.

**Table 3:** Primary estimand for the STOMP/UNITY conjoined analysis.

Primary Objective: To compare the clinical efficacy of tecovirimat versus placebo in participants with HMPXV.	
Estimand	The percent decrease in time to clinical resolution [complete healing] of skin or visible mucosal lesions among people aged 18 or older with laboratory-confirmed mpox with 1 or more skin or visible mucosal lesions in those prescribed tecovirimat ± available rescue medication as needed relative to those prescribed no treatment ± available rescue medication as needed
Treatment	Treatment of interest: Tecovirimat for 14 days
	Alternative treatment to which comparison will be made: No treatment
Target population	Analysis set
People aged 18 or older with laboratory-confirmed HMPXV disease of <14 days duration with 1 or more skin or visible mucosal lesions	Primary Efficacy Set, defined as all randomised participants with laboratory-confirmed mpox, one or more skin or visible mucosal lesions, and <14 days of symptoms prior to study entry
Variable(s)	Outcome measure(s)
Time to clinical resolution up to 28 days	Time to clinical resolution is measured from randomisation to the first day or visit on which all skin and visible mucosal lesions are scabbed, desquamated, or healed, up to 28 days after randomisation (Day 29)
Time to complete healing up to 28 days	Time to complete lesion healing is measured from randomisation to the first day or visit on which all skin and visible mucosal lesions are healed with a new layer of skin re-epithelialization, up to 28 days after randomisation (Day 29)
Handling of intercurrent events	Handling of missing data
• All-cause death Follow-up until the time of death will be included (While-on-treatment strategy) • Treatment change due to disease progression or severe pain Follow-up through 28 days will be included, regardless of the use of open-label tecovirimat (Treatment policy strategy) • Use of other antivirals with expected activity against HMPXV/rescue medications Follow-up through 28 days will be included, regardless of the use of other antivirals/rescue medications (Treatment policy strategy) • Blinded treatment discontinuation for any other reason Follow-up through 28 days will be included, regardless of treatment status (Treatment policy strategy)	Participants who are lost to follow up will be censored at time of last lesion evaluation.
Population-level summary measure	Analysis approach
Shortening, in percentage, of time to clinical resolution [complete healing] for tecovirimat ± available rescue medication as needed relative to no treatment ± available rescue medication as needed	The percent decrease in time to clinical resolution [complete healing] will be estimated with the associated 95% confidence interval and tested using a parametric accelerated failure time model stratified by trial (i.e., STOMP or UNITY) and time from symptom onset (≤7 vs. >7 days)

## Outstanding questions

Several important questions remain regarding the design and implementation of federated trials. These include how best to handle heterogeneity of treatment effects across trials; how to design interim monitoring and multiplicity adjustments that remain transparent when trials evolve at different speeds during an emergency; and which governance, funding, and contractual models most reliably support a shared DMC and data-sharing infrastructure across independent sponsors.

## Conclusion

The one-trial, one-protocol, one-sponsor approach should remain the gold standard. But the federated trials approach has advantages in settings that present major obstacles to conducting that classic single-trial design. It lets academic sponsors run separate trials with geographical, organisational, or logistical limitations while planning strong, rigorous regulatory collaboration across trials. The federated design makes it possible to do conjoined analyses during and after trials. Investigators predetermine the joint sample size and draft the joint SAP that those conjoined analyses will use before individual trials launch. The federated approach also distributes efforts and responsibilities, making organisations leaner and more flexible, critical attributes in dynamic situations like pandemics. It also reduces uncertainty in emerging outbreak scenarios where making accurate forecasts is extremely challenging. Finally, the federated trial design makes it possible to implement trial results quickly.

But this approach has disadvantages compared to the classic single-trial design, including variation between protocols. It is also more difficult to achieve the robust and adequately controlled evidence of treatment effect required to apply for marketing authorisation of novel drugs. Nonetheless, investigators can overcome these problems by forming strong collaborations and focussing on methodology and scientific rigour. The optimal approach should depend on whether a single, globally coordinated trial is feasible in a public-health emergency or other crises. Running one large trial to completion provides more robust results than running multiple smaller trials, even when they produce a conjoined analysis.

Our discussions with regulators showed they clearly prefer single trials for consistency and robustness, but they also acknowledged the challenges academic institutions face when setting up trials to bring new drugs to market under public-health emergencies. Regulators remain open, in exceptional circumstances, to approving market authorisations based on a conjoined analysis.

Practical considerations are also critical for the success of federated trials. As the MPX-RESPONSE consortium showed, forming a common DMC and shared analytic infrastructure may require dedicated funding mechanisms and clear governance agreements across sponsors.

Another advantage of the federated approach is that it explicitly acknowledges and addresses regional differences in trial conduct, such as variations in clinical practice, capacity or standards for supportive treatment. Large, international, single-protocol trials often treat these variations as implicit and do not address them. The prospective, proactive strategy of federated trials strengthens the interpretability and the credibility of the pooled evidence they generate.

Any federated trials design, like an adaptive trials design, requires a clear justification for why standard approaches such as a single RCT are suboptimal. Critical elements of this justification are (1) a scientific rationale, (2) how the proposal addresses regulatory needs, (3) how to ensure the integrity of trials as they are implemented, (4) type I error control, (5) reducing bias (e.g., from selecting ongoing trials with interim analyses), and (6) the practicality and feasibility of the proposal.^25^ Investigators also need to state the rationale for conducting multiple trials, such as geographic, political, or regional reasons.

Our discussion draws on two complementary examples: the MPX-RESPONSE consortium and the UNITY/STOMP collaboration. The former demonstrates a functioning federated design while the latter highlights the opportunities and limitations of extending such coordination to trials initiated independently through the conjoined analyses. Using both examples helps us explore the methodological boundaries of the federated trial framework.

Federated trials build on the growing plethora of meta-analytic methods, such as standard retrospective aggregate data or individual patient data meta-analyses, prospective meta-analyses, or meta-trial designs. The main difference between these methods and the federated approach is the strict regulatory requirements for using data to request marketing authorisation. This is a novel approach that to our knowledge has been applied rarely and never with multiple sponsors.^26^

Public health emergencies require new solutions that are flexible but scientifically rigourous, and the federated trials design offers investigators one such solution. It represents another tool for generating timely, robust evidence and providing patients with new treatment options that are safe and effective as quickly as possible.

## Contributors

The authors contributed to the trial design (MPX-RESPONSE trials: ICO, SH, AA, YY, MB; STOMP: LZ) and the discussions between UNITY and STOMP for the conjoined analysis (ICO, LZ, SH, AA, YY, MB). TRF was part of the WHO CORE protocol writing team and provided valuable insights on this process. FK contributed with regulatory insights. All authors provided input to and have approved the final version of the manuscript.

## Data sharing statement

Not relevant.

## Declaration of interests

ICO declares other funding by the South-East Norway Regional Health Authority, consulting fees from Dilafor AB and Simplexia AB, support for attending meetings from European Medicines Agency and the European Clinical Research Infrastructure Network and participation in several Data Safety Monitoring Boards at Oslo University Hospital; LZ, SH, AA, YY, FK, TRF, and MB declares no relationships, activities or other interests related to the content of this manuscript other than the funding stated in the acknowledgements.

## Appendix A Supplementary data

Supplementary Material

List of Group Authors for UNITY and STOMP
