# Supplementary material for: The federated trials approach; an opportunity for global collaboration in health emergencies
Source: eClinicalMedicine. 2026 Feb 25;93:103809. doi: 10.1016/j.eclinm.2026.103809 (PMC12955581; doi:10.1016/j.eclinm.2026.103809)
Supplement: Supplementary Material [file mmc1.pdf]

MPX-RESPONSE

A clinical research network to improve the management of Mpox virus disease

Data Safety and Monitoring Board (DSMB) Charter

Version 1.1, 25 October 2023

Authorised by:

Name:	Rob Fowler	Role:	Chair DSMB
Signature:		Date:	

Authorised by:

Name:	Yazdan Yazdanpanah	Role:	Chair MPX-RESPONSE Executive Committee
Signature:		Date:	

Authorised by:

Name:	Alexandra Calmy	Role:	PI UNITY Switzerland
Signature:		Date:	

Authorised by:

Name:	Beatriz Grinsztejn	Role:	PI UNITY Brazil
Signature:		Date:	

Authorised by:

Name:	Miquel Ekkelenkamp	Role:	PI EPOXI
Signature:		Date:	

DSMB CHARTER FOR MPX-RESPONSE

CONTENT<br>Guidance	CHARTER DETAILS
**1. Introduction**	
Name of DSMB	MPX-RESPONSE Data Safety and Monitoring Board
Objectives of the project	The MPX-RESPONSE project aims to conduct clinical trials for mpox therapeutics to support better treatment of mpox disease and further inform public health policy and clinical management.<br><br>MPX-RESPONSE will endeavour to: (i) assess clinical and virological outcomes of treatments against mpox in a clinical cohort in Europe, focusing on severe cases where clinicians would be reluctant to randomised controlled trials with placebo arms. (ii) determine the effect of different treatment and management options on the clinical outcomes of human mpox infection in randomized controlled trials that will mostly target mild and moderate patients.
Common framework and core protocols	The MPX-RESPONSE project has received funding from the Horizon Europe Programme (grant number 101115188), and a consortium has been established based on this project.<br><br>All randomised clinical trials under the MPX-RESPONSE umbrella will be based on the WHO CORE protocol for adaptive multiregional international global randomized, placebo-controlled trials to evaluate the safety and efficacy of drugs for the treatment of human mpox (https://cdn.who.int/media/docs/default-source/blue-print/final-core-protocol-monkeypox-therapeutics_25-july-2022.pdf?sfvrsn=ac660454_3&download=true).<br><br>A common core protocol enables a strong collaboration between the trials, including a common DSMB. The MPX-RESPONSE consortium has initiated some core facilities to help out the individual trials, such data management, statistics and pharmacovigilance. Support for the common DSMB is maintained in the Committee Liaison and Coordination (CLC) Secretariat, which also supports other committees of the MPX-RESPONSE consortium. Coordinators of the CLC includes Erica Telford (erica.telford@inserm.fr) and Nicolas Pulik (nicolas.pulik@anrs.fr).<br><br>Note that in this charter, a trial is understood as an investigation under a common protocol and governed by a single Trial Steering Committee (TSC). Each trial can have one or several sponsors. Details of each trial is given in Annexe 3.
Data sharing with DSMB committees of other mpox clinical trials	Due to the nature of the current mpox epidemic, with unprecedented spread outside of Africa reported since May of 2022, it is foreseen that health emergencies may urge to data sharing between DSMBs of trials outside the MPX-RESPONSE consortium on treatment of mpox

**CONTENT**<br>Guidance	**CHARTER DETAILS**
	infection. Data may be shared at the discretion of the DSMB, with prior consent of the Trial Steering Committees of the trials and with appropriate (contractual) safeguards to guarantee confidentiality of the data, as long as this is in the interest of the MPX-RESPONSE trials specifically and mpox patients in general.<br><br>The DSMB may be asked to review external evidence and data from other mpox trials.
Outline of scope of charter	The purpose of this document is to describe the roles and responsibilities of the common independent DSMB for the MPX-RESPONSE trials, including the timing of meetings, methods of providing information to and from the DSMB, frequency and format of meetings, statistical issues and relationships between the trials within and outside MPX-RESPONSE.
**2. Roles and responsibilities**	
Aims of this committee	The DSMB, common to all countries, sites and participants in the MPX-RESPONSE trials, will monitor accumulating efficacy and safety data on an ongoing basis. The mission of the DSMB will be to safeguard the interests of trial participants and to enhance the integrity and credibility of the trial. The DSMB will be asked to recommend stopping early for efficacy only when there is clear and substantial evidence of a treatment benefit. Details of formal stopping rules will be given for each trial in Annexe 3.
Terms of reference	The DSMB should receive and review information on the progress and accruing data of the trials and provide advice and recommendations on the conduct of the trial to each of the Trial Steering Committees (TSCs). The TSCs, as decision-making committees of the trials, will validate the proposed choices. For the sake of clarity, the DSMB is only advisory and formal decisions are made in the TSCs.<br><br>This charter outlines the general principles for the DSMB. Trial-specific requirements, such as specific stopping rules, are detailed in the Annexe 3. Note that it is possible for the DSMB to recommend stopping an investigation in one trial and continuing the same investigation in another trial if this is in the interest of the stakeholders of the individual trial.<br><br>It is expected from each trial and each sponsor that data can be shared between trials such that recommendations can be made based on all available information.
Specific roles of DSMB	Interim review of the trials' progress including updated figures on recruitment, data quality, adherence to protocol treatment and follow-up, and main outcomes and safety data. Specifically, these roles include to:<br>- monitor evidence for trial safety (e.g. toxicity, SAEs and SUSARs, deaths)<br>- assess the impact and relevance of the mpox emergency new data

**CONTENT**<br>Guidance	**CHARTER DETAILS**
	• decide whether to recommend that a trial continues to recruit participants or whether recruitment should be terminated either for everyone or for some treatment arms and/or some participant subgroups<br>• decide whether to recommend that trial follow-up should be stopped earlier<br>• assess data quality, including completeness<br>• maintain confidentiality of all trial information that is not in the public domain<br>• monitor recruitment figures and losses to follow-up<br>• monitor compliance with the protocol by participants and investigators<br>• suggest additional data analyses, if necessary<br>• review proposed protocol changes for potential harm to the patient<br>• monitor compliance with previous DSMB recommendations<br><br>The DSMB may also be asked to review external evidence and individual patient data from other mpox trials, if it is in the interest of the MPX-RESPONSE trials specifically and mpox patients in general.
**3. Before or early in the trial**	
Input into the protocol	All potential DSMB members should have sight of all protocols before agreeing to join the committee. Therefore, if a potential DSMB member has major reservations about any of the trials (e.g. the protocol or the logistics) they should report these to the Chair of the relevant TSC and may decide not to accept the invitation to join. DSMB members should be independent[1] and constructively critical of the ongoing trials, but also supportive of aims and methods of the trials.
Whether the DSMB will meet at the beginning of the trial	At the initiative of the TSCs, the DSMB will first meet early in the course of the trials, to discuss the protocol, the trials, the analysis plans, future meetings, and to have the opportunity to clarify any aspects with the TSC chairs, Chief Investigators or other core functionalities (statistics, pharmacovigilance, data management etc).<br>Consideration shall be given to an initial "dummy" report, including the use of shell (empty) tables, to familiarise the DSMB members with the format that will be used in the reports.
Any other issues specific to the treatment under study	In the late spring and early summer, an unprecedented epidemic of human mpox infection has been experienced, particularly affecting men who have sex with men (MSM). This outbreak has been the underlying reason to conduct the MPX-RESPONSE trials. However, likely due to general awareness and public health measures, patient numbers in the epidemic have rapidly declined, which will have an impact on patient recruitment. On the other hand, the possibility that the epidemic will resurge needs to be considered, which will be the

[1] Independence is defined in the table in Annexe 1

CONTENT<br>Guidance	CHARTER DETAILS
	reason for organizing the trials, even when patient numbers are low. This background will need to be taken into consideration by the DSMB when analysing trial progress and recommendations for (dis)continuation of the trial(s).<br><br>The first treatment under investigation will be tecovirimat. It should be noted that the authorisation parameters used by health authorities for tecovirimat are exceptional, as smallpox is eradicated and mpox was mainly limited to specific geographical areas until 2022. Tecovirimat has never been tested in humans in randomised controlled clinical studies, efficacy data were obtained in animal models, and limited safety and pharmacodynamics/pharmacokinetics studies were performed in humans.<br><br>As new or repositioned therapeutic options for mpox treatment are studied and show a sufficient safety profile for phase 3 evaluation, they may be added to the trials as separate therapeutic arms, according to control and randomisation rules described in each trial's protocol.<br><br>Standard of care provided to patients will be adapted in each site to recommendations of local health authorities.
Whether members of the DSMB will have a contract	DSMB members will not formally sign a contract for their role in the DSMB. They should formally register their agreement to join the group by confirming (1) that they agree to be on the DSMB (2) that they agree with the contents of this Charter, and (3) they should disclose any potential competing interest which may be perceived by some as preventing them from making decisions to the benefit of the trial. **Members should complete and return the form in Annexe 2 before accepting the invitation to join the DSMB**. Any changes in the registered conditions by a member of the DSMB should be immediately communicated to the CLC Secretariat.<br>Observers attending any part of the meeting should sign a confidentiality agreement on the first occasion they attend all or part of a meeting (Annexe 2).<br><br>The Curriculum Vitae of the DSMB members must be systematically collected for the setting up of the committees.
**4. Membership**	
Membership and size of the DSMB	The DSMB will be composed of four to six voting members, as far as possible internationally distributed. At least three members including the Chair and the independent statistician will constitute a quorum. The members are experts in clinical trials and in clinical medicine, pharmacology, and statistics.<br><br>One of the voting DSMB members will be a statistician or a methodologist to provide independent statistical expertise, especially with regards to interpretation of accumulating data and guidance through the report.

**CONTENT**<br>Guidance	**CHARTER DETAILS**
	The trial DSMB members are listed in Appendix 1.<br><br>The Chairs of the TSCs approve the composition of the DSMB and appoint the members.<br>The members should be independent of the trial (should not be involved with the trial in any other way or have any competing interest(s) that could impact on the trial). Any competing interests, both real and potential, should be declared through the form in Annexe 2.
The Chair, how they are chosen and the Chair's role.	The Chair will have previous experience of serving on DSMBs, experience of Chairing meetings and will be able to facilitate and summarise discussions. The Chair is appointed by the TSCs.
The responsibilities of the DSMB statistician	The DSMB will include a statistician or a methodologist to provide independent statistical expertise, especially with regards to interpretation of accumulating data and guidance through the report. The DSMB statistician will not prepare the DSMB report.
The responsibilities of the trial statistician	The trial statistician of each trial will have overall responsibility for the statistical aspects of the corresponding trial, including the pre-specification of any formal stopping rules for efficacy. The trial statistician will prepare the statistical analysis plan and perform the final analyses for the trial, and participate in the open part of DSMB meetings, participating in DSMB discussions on the open report and, on some occasions, taking notes.<br>Data collection, data management, and data quality assurance for the trial will be carried out by the trial dedicated working groups.
The Responsibilities of unblinded independent statistician	The unblinded independent statistician(s) will be non-voting member of the DSMB. Primary responsibilities of the unblinded independent statistician(s) are:<br>- Provide tables, listings and figures for both the open and the closed reports to the DSMB<br>- Provide a summary for closed reports that interprets the statistical analyses as part of the closed report<br>- Produce the close report summary<br>- Provide ad hoc analyses, as needed<br>- Distribute the closed reports to the DSMB<br>- Participate in the open and closed sessions of all DSMB meetings<br>- Present analyses to the DSMB during closed sessions of the DSMB<br>- The reports should contain both aggregate data across all trials and trial specific information according to each trial's requirements (see Annexe 3). A sample (dummy) report will be

**CONTENT**<br>Guidance	**CHARTER DETAILS**
	presented to the trial statisticians prior to the first meeting for review.<br>The unblinded independent statistician(s) will be contracted from an organisation outside the MPX-RESPONSE consortium.<br><br>Summaries will be made by each trial separately and in combination when the treatment arms are comparable 1 week (5 business days) before each meeting.<br><br>Each trial may nominate one unblinded independent statistician. This can be the same statistician.
The responsibilities of the Statistical and data management working group	The Project Manager and/or other members of this group may attend open sessions of the meeting.
The responsibilities of the coordinating investigator, co-PI and other members of the Trial Steering Committee (TSC)	The coordinating investigators and the co-PIs may be asked, and should be available, to attend open sessions of the DSMB meeting. The other TSC members will not usually be expected to attend but can attend open sessions when necessary.
**5. Relationships**	
Relationships with the other trial committees (e.g. Trial Steering Committee, TSC; Trial Management Team, TCT; Committee Liaison & Coordination Secretariat, CLC Secretariat)	The TSCs oversee the trials. The DSMB is advisory to the TSCs. The DSMB make comments, requests, and recommendations to the TSCs, who will consider them for final decisions.<br><br>All communications between the TSCs and the DSMB committees will be facilitated by the CLC Secretariat.
Payments to DSMB members	Members will be reimbursed for reasonable travel costs and accommodation where required. No other payments or rewards are given.
**6. Organisation of meetings**	
Expected frequency of DSMB meetings	Details of the meeting frequency is given in the Annex 3.<br>Note that reason should applied when deciding meetings and meeting frequency, taking into consideration the request from each trial while keeping the meeting intervals reasonable. Extraordinary meetings may be requested by the trials central Safety officer in case of unexpected or serious issues including protocol defined new safety issues and unanticipated problems.
Whether meetings will be face-to-face or by teleconference	The DSMB meets either in presence or by teleconference, although the latter will be preferred to ensure reactivity. Meetings are organised by CLC Secretariat.

**CONTENT**<br>Guidance	**CHARTER DETAILS**
How DSMB meetings will be organised, especially regarding open and closed sessions	A mixture of open and closed sessions will be held. Only DSMB members, the unblinded statisticians and others whom they specifically invite in the close session, e.g the trials central Safety Officers, might be invited in closed sessions for data safety review and expertise. In open sessions, all those attending the closed session may be joined by relevant representatives as detailed below.<br><br>The format of the meetings will be based on the following structure:<br><br>1. Open session – Introduction and any disclosed parts of the report: during the open session, only blinded material will be reviewed. The open session of the DSMB will allow time for the TSC (coordination investigator, co-PI, trial statistician, trial data management and experts according to the agenda of the meeting) to discuss study status and bring up any safety concerns in which they would like input from the DSMB. This session will also be used to discuss amendments to the protocol, Investigator's Brochure if applicable or SmPC and/or external data relevant to the trial.<br><br>The open session will be attended by the Chairs of the TSCs, the CLC Secretariat, PIs and Co-PIs, trial statisticians and Safety Officers. If relevant, others might be invited, such as representatives of funders or regulators, data managers, lead monitors etc.<br><br>2. Closed session: during the DSMB closed session, data summaries for each trial by arms will be presented by the unblinded statistician. The data presentation will be unblinded.<br><br>The closed session will be attended by all voting members of the DSMB, the unblinded independent statistician and when judged necessary invited experts or the unblinded Safety Officer. All data presented in the closed report, or during the closed session, and all discussion during the closed session will be confidential.
**7. Trial documentation and procedures to ensure confidentiality and proper communication**	
Intended content of material to be available in open sessions	Accumulating information relating to recruitment and data quality (e.g. data return rates, treatment compliance) along with baseline characteristics will be presented. Toxicity details and outcome measures based on data pooled across treatment groups may be presented at the discretion of the DSMB.
Intended content of material to be available in closed sessions	In addition to all the material available in the open session, the closed session material will include outcomes developed in each trial and combined, divided by the randomly allocated treatment.
The people who will see the accumulating data and interim analysis	The accumulating data and interim analysis on safety data and outcomes will be seen by the DSMB members and unblinded independent statistician.

**CONTENT**<br>Guidance	**CHARTER DETAILS**
Responsibility for identifying and circulating external evidence (e.g. from other trials/systematic reviews)	Identification and circulation of external evidence (e.g. from other trials/systematic reviews) is not the responsibility of the DSMB members. The CLC Secretariat will collate any such information for presentation in an open session.
To whom the DSMB will communicate the decisions/ recommendations that are reached	The DSMB communicate its recommendations in writing to the TSCs, through a report addressed to the Chairs of the for information and decision. The CLC Secretariat will also receive the report for information.
Whether reports to the DSMB be available before the meeting or only at/during the meeting	The DSMB should receive the interim report at least 72 hours before a meeting and preferably earlier if time allows due to unusual circumstances. In case of unexpected efficacy or any safety concern, immediate communication to sponsor and trials' TSCs should be made before receiving the minutes.
**8. Decision making**	
What decisions/recommendations will be open to the DSMB	Possible recommendations from the DSMB include:<br>- Monitoring of safety and outcomes<br>- Proposing or commenting on proposed protocol changes<br>- No action needed, trial continues as planned<br>- Commenting on Statistical Analysis Plan (applicable before the unblinded data being seen by the DSMB)<br>- Propose any recommendation on the following:<br>1. Analysis of benefit or harm of a treatment, clear lack of benefit or external evidence.<br>2. Analysis of trial subgroups (care should be taken if this is not a pre-specified subgroup).<br>3. Extending recruitment (based on actual control arm response rates being different to predicted rather than on emerging differences)<br>4. Extending follow-up<br><br>Separate recommendations for each trial and TSC according to specifications in each trial protocol might occur.
How recommendations will be reached within the DSMB	The DSMB Chair is to summarise discussions and encourage consensus; it is usually best for the Chair to give their own opinion last.<br>Every effort should be made for the DSMB to reach unanimous recommendations. If the DSMB cannot achieve this, a vote may be taken, although details of the vote <u>should not</u> be routinely included in the report addressed to the TSC as these may inappropriately convey information about the state of the trial data. If the vote is tied, the recommendation will be made according to the opinion of the Chair.<br>It is important that the implications (e.g. ethical, statistical, practical, and financial) for the trial be considered before any recommendation is made.

**CONTENT**<br>Guidance	**CHARTER DETAILS**
	The DSMB Chair will appoint a minute taker among the closed session participants. The minutes will be circulated after the meeting and finalised/approved before the recommendations are sent to the TSCs. The minutes will be kept confidential until the trials are closed.
When the DSMB is quorate for recommendation-making	Efforts should be made to ensure that all members can attend. The CLC Secretariat will try to ensure that a date is chosen to enable this. If, at short notice, any DSMB members cannot attend at all then the DSMB may still meet if at least one statistician and one clinician, including the Chair (unless otherwise agreed), will be present. If the DSMB is considering recommending major action after such a meeting, the DSMB Chair should communicate with the absent members as soon after the meeting as possible to confirm their agreement. If they do not, a further meeting should be arranged with the full DSMB.
Can DSMB members who cannot attend the meeting give input	If the report is circulated before the meeting, DSMB members who will not be able to attend the meeting may pass comments to the DSMB Chair for consideration during the discussions.
**9. Reporting**	
To whom will the DSMB report their recommendations and decisions, and in what form	Recommendations from the DSMB will be reported through a recommendation letter to the TSCs for decision consideration and, for information, to the CLC Secretariat, and the representatives of Sponsors. If the recommendations are equal for all trials and TSCs then a single letter is sufficient. If there are differences in the recommendations then separate letters to each TSC is required.<br><br>If their recommendation is that the trial should continue unchanged, then a letter should be sent to the aforementioned recipients usually 72 hours after the meeting. If their recommendation is to change the trial design or stop the trial all together, then a letter should be sent to the aforementioned recipients and within 24 hours.

Abbreviations and glossary

CLC	Committee Liaison & Coordination
CRF	Case Reporting Form
DSMB	Data Safety and Monitoring Board
SAE	Serious Adverse Event
SUSAR	Suspected Unexpected Serious Adverse Reaction
TCT	Trial Coordination Team
TSC	Trial Steering Committee

Annexe 1: List of members of the Data Safety and Monitoring Board (DSMB)

Dr. Rob Fowler, Sunnybrook Health Sciences Centre, Toronto, ON., Canada (Chair)

Prof. Cristina Mussini, University of Modena and Reggio Emilia, Modena, Italy

Prof. David DeMets, School of Medicine and Public Health, University of Winsconsin-Madison, Madison, WI., USA

Dr. Olavo Henrique Munhoz Leite, University of São Paulo, São Paulo, Brazil

Dr. Antoinette Tshefu, Kinshasa School of Public Health, Democratic Republic of the Congo (DRC)

Annexe 2: Agreement and potential competing interest declaration form

UNITY: Agreement to join the Data Safety Monitoring Board and disclosure of potential competing interests

Please complete the following document and return to the Committee Liaison and Coordination (CLC Secretariat.

(Please tick box to agree)

☐	I have read and understood the DSMB Charter version 1.0, dated 16 February 2023, and agree with its content
☐	I agree to join the Data Safety Monitoring Board for the MPX-RESPONSE trials
☐	I agree to treat all sensitive trial data and discussions confidentially*

**Checking this box implies agreeing also with the statement that DSMB members should not use any trial data to inform trading in pharmaceutical shares, and careful consideration should be given to trading in stock of companies with competing products.*

The avoidance of any perception that members of a DSMB may be biased in some fashion is important for the credibility of the decisions made by the DSMB and for the integrity of the trial.

Possible competing interest should be disclosed via the CTU. In many cases, simple disclosure up front should be sufficient. Otherwise, the (potential) DSMB member should remove the conflict or stop participating in the DSMB. **Table 1** lists potential competing interests.

☐ **No,** I have no competing interests to declare
☐ **Yes,** I have competing interests to declare (please detail below)

Please provide details of any competing interests:

____

____

____

Name: ____________________________

Signed: ___________________________ Date: ______________

Table 1: Potential competing interests

☐ Stock ownership in any commercial companies involved
☐ Stock transaction in any commercial company involved (if previously holding stock)
☐ Consulting arrangements with the Sponsor/Funder
☐ Ongoing advisory role to a company providing drugs to the trial
☐ Frequent speaking engagements on behalf of the intervention
☐ Intellectual conflict e.g. strong prior belief in the trial's experimental arm
☐ Involvement in regulatory issues relevant to the trial procedures
☐ Investment (financial or intellectual) or career tied up in competing products

Annexe 2: Agreement and confidentiality agreement for observers

UNITY: Agreement to attend the Data Safety Monitoring Board meeting and treat all information confidentially

Please complete the following document and return to the Committee Liaison and Coordination (CLC Secretariat.

(Please initial boxes to agree)

	I have received a copy of the DSMB Charter version 1.0 16 February 2023
	I agree to attend the DSMB meeting on ___/____/______
	I agree to treat as confidential any sensitive trial information gained during this meeting unless explicitly permitted

Name: ____________________________

Signed: ___________________________ Date: _______________

Annexe 3: Trial-specific specifications to the DSMB charter

Currently there are two trials under the MPX-RESPONSE project, the UNITY and the EPOXI trial.

The EPOXI trial:
This multi-country randomized, placebo-controlled, double-blinded trial will evaluate the safety and efficacy of tecovirimat treatment (600 mg q12h, 14 days, orally) for adult patients with mpox virus disease. The trial runs in several countries in the European Union and the European Economic Area (EU/EEA) under the EU Clinical Trial Regulation. There is a single sponsor (University Medical Center Utrecht) in this trial.
The current approved protocol is version 3.0 dated 05 October 2023.

The overall objective of the study is to evaluate the clinical efficacy, as assessed by time to lesion resolution, of treatment plus standard of care for patients with mpox. The primary endpoint is time for all lesions (skin or mucosal) to heal with a new fresh layer of skin "re-epithelialization" (i.e., resurfacing of a wound with a new epithelium layer).
Reporting of the endpoints, including the primary endpoint, will be descriptive in nature, since the projected number of inclusions (150) will yield insufficient statistical power to demonstrate superiority of the intervention.

Key secondary outcomes reported to the DSMB will be (1) Time to complication or all-cause admission to hospital or all-cause death, including a frequency table of types of complications, and (2) time to resolution of pain. (see description in the study protocol paragraph 13.6.2).
Safety outcomes will include an overview of All-cause mortality within 28 days and within 90 days and AEs, SEAs and SUSARs (see description in the trial protocol paragraph 13.6.2).

Meeting frequency:
The DSMB will review safety data after the first 100 subject are enrolled and have been followed for 15 days. Other reports will follow at regular intervals and after every extra 200 patients have been followed for 15 days. Note that the numbers refer to the combined number of patients in all the MPX-RESPONSE trials, and the review will be on the combined data.

Recommendation rules:
According to the current protocol, stopping for efficacy should only be recommended when there is a clear and substantial evidence of a treatment benefit. The threshold for stopping will be high, i.e. at a p-value of 0.001 for rejecting the null hypothesis of no treatment effect on the primary endpoint. An interim assessment of efficacy will be done after approximately 300 subjects have been followed for 15 days. The final analysis after 500 randomized patients will adjust for the alpha spent at the interim analysis. Note that the numbers and analyses (both for efficacy and safety) are based on the combined data from all the MPX-RESPONSE trials.

The Unity trial:
This international adaptive multi-country randomized, placebo-controlled, double-blinded trial will evaluate the safety and efficacy of treatments for adult and paediatric (>13 Kg of weight) patients with mpox virus disease. In the current version of the protocol a single intervention is investigated (tecovirimat 600 mg q12h, 14 days, orally vs matching placebo). There is a separate sponsor in each country the trial is running. This sponsor is responsible for the initiation, management, and financing of the trial in their country. The MPX-RESPONSE has core facilities to help out with the management such as a common data management, statistics and pharmacovigilance. There will be a single, common TSC governing the trial on behalf of all the sponsors.

The current approved protocol is version 2.0 dated 13 October 2022

The overall objective of the study is to evaluate the clinical efficacy, as assessed by time to lesion resolution, of treatment plus standard of care (SoC) for patients with mpox.

- The primary endpoint is time for all lesions (skin or mucosal) to heal with a new fresh layer of skin "re-epithelialization" (i.e., resurfacing of a wound with a new epithelium layer).

Secondary outcome measures include:
- To evaluate the clinical efficacy of treatment plus SoC versus placebo plus SoC in patient with mpox as assessed by mortality, clinical severity, and duration of symptoms
- To evaluate safety of treatment plus SoC relative to placebo plus SoC in patients with mpox

Meeting frequency:

The DSMB will review safety data after the first 100 subject are enrolled and have been followed for 15 days. Other reports will follow at regular intervals and after every extra 200 patients have been followed for 15 days. Note that the numbers refer to the combined number of patients in all the MPX-RESPONSE trials, and the review will be on the combined data.

Recommendation rules:
According to the current protocol, stopping for efficacy should only be recommended when there is a clear and substantial evidence of a treatment benefit. The threshold for stopping will be high, i.e. at a p-value of 0.001 for rejecting the null hypothesis of no treatment effect on the primary endpoint. An interim assessment of efficacy will be done after approximately 300 subjects have been followed for 15 days. The final analysis after 500 randomised patients will adjust for the alpha spent at the interim analysis. Note that the numbers and analyses (both for efficacy and safety) are based on the combined data from all the MPX-RESPONSE trials.

Annexe 4: Summary of changes from previous versions

Version 1.0

This is version 1.0 of the DSMB charter for this trial and is numbered this way for historical reasons. This is the first version of the DSMB charter, so there are no changes to be reported from previous versions.

Version 1.1

Due to an updated protocol in EPOXI, the Annexe 3: Trial-specific specifications to the DSMB charter has been updated with new specifications for EPOXI.
