# Supplementary material for: The federated trials approach; an opportunity for global collaboration in health emergencies
Source: eClinicalMedicine. 2026 Feb 25;93:103809. doi: 10.1016/j.eclinm.2026.103809 (PMC12955581; doi:10.1016/j.eclinm.2026.103809)
Supplement: List of Group Authors for UNITY and STOMP [file mmc2.docx]

# List of Group Authors for UNITY/MPX-RESPONSE and STOMP Study Groups

## UNITY/MPX-RESPONSE Study Group

| First Name | Surname | Country |
| --- | --- | --- |
| Beatriz | **Grinsztejn** | Brazil |
| Valdiléa G. | **Veloso** | Brazil |
| Sandra Wagner | **Cardoso** | Brazil |
| Mayara | **Secco Torres da Silva** | Brazil |
| Maria Pia | **Diniz** | Brazil |
| José Valdez | **Ramalho Madruga** | Brazil |
| Jorge | **Andrade Pinto** | Brazil |
| Esaú | **Custódio João** | Brazil |
| Luiz Carlos | **Pereira Junior** | Brazil |
| José Henrique | **Pilotto** | Brazil |
| Carlos | **Brites** | Brazil |
| Maria Paula | **Gomes Mourão** | Brazil |
| Alexandra | **Calmy** | Switzerland |
| Maxime | **Hentzien** | Switzerland |
| Olivier | **Segeral** | Switzerland |
| Stefano | **Musumeci** | Switzerland |
| Laurence | **Toutous-Trellu** | Switzerland |
| Sabine | **Yerly** | Switzerland |
| Benjamin | **Hampel** | Switzerland |
| Pedro Enrique | **Cahn** | Argentina |
| Maria José | **Rolón** | Argentina |
| Dimie | **Ogoina** | Nigeria |
| Miguel | **Ekkelenkamp** | The Netherlands |
| Nathalie | **Strub-Wourgaft** | France |
| Ines | **Aristegui** | Argentina |
| Thiago | **Torres** | Brazil |
| Osilade | **Adewole** | Nigeria |
| Roger | **Lewis** | USA |
| Yazdan | **Yazdanpanah** | France |
| France | **Mentré** | France |
| Axelle | **Dupont** | France |
| Najeh | **Daabek** | France |
| Inge Christoffer | **Olsen** | Norway |
| Skerdi | **Haviari** | France |
| Cedric | **Laueuenan** | France |
| Alain | **Amstutz** | Switzerland |
| Matthias | **Briel** | Switzerland |
| Constance | **Delaugerre** | France |
| Xavier | **de Lamballerie** | France |
| Gilles | **Peytavin** | France |
| Alpha | **Diallo** | France |
| Léa | **Vitu** | France |
| Mario | **DELGADO-ORTEGA** | France |
| Erica | **TELFORD** | France |
| Keyla | **DE ALMEIDA MACEDO** | France |
| Roberta | **Trefiglio** | Brazil |
| Laetitia | **GUIRAUD** | Switzerland |
| Luciana | **Gambardella** | Argentina |
| Carolina | **Perez** | Argentina |
| Ventzislava | **Petrov-Sanchez** | France |
| Michèle | **Genin** | France |
| Maelle | **Coupez** | France |
| Isabelle | **HOFFMANN** | France |
| Cecilie | **Moe** | Norway |
| Maria | **Figueroa** | Argentina |

**STOMP Study Group**

| First Name | Surname |
| --- | --- |
| Timothy | **Wilkin** |
| William | **Fischer** |
| Jason | **Zucker** |
| Lara | **Hosey** |
| Jhoanna | **Roa** |
| Arzhang | **Javan** |
| John | **Brooks** |
| Judith | **Currier** |
| Joseph | **Eron** |
| Rajesh | **Gandhi** |
| Matthew | **Hamill** |
| Kieron | **Leslie** |
| Sharon | **Nachman** |
| Caitlyn | **McCarthy** |
| Carlee | **Moser** |
| Justin | **Ritz** |
| Pooja | **Saha** |
| Lu (Summer) | **Zheng** |
| Stephanie | **Caruso** |
| Caroline | **Reeb** |
| Shahadah | **Bailey** |
| Lauren | **Mabe** |
| Lisette | **Molins** |
| Sujith | **Valiyaparambil** |
| Grace | **Aldrovandi** |
| Kathie | **Ferbas** |
| Faye | **Landsman** |
| Jade | **Paris** |
| Danielle | **Campbell** |
| Stanford | **Chimutimunzeve** |
| Kristina | **Brooks** |
| Edmund | **Capparelli** |
| Shawn | **Chiambah** |
| Jonathan | **Berardi** |
| Alexander L. | **Greninger** |
| Christine | **Johnston** |
| David | **Smith** |
| Cheryl | **Day** |
| Emily | **Blum** |
| Josie | **Marshall** |
| Jillian | **Laroche** |
| Bridget | **Makhlouf** |
